# Nonlinear Forced Response of Electromechanical Integrated Toroidal Drive to Coupled Excitation

**DOI:** 10.1100/2012/743138

**Published:** 2012-09-25

**Authors:** Lizhong Xu, Fen Wang

**Affiliations:** Mechanical Engineering Institute, Yanshan University, Qinhuangdao 066004, China

## Abstract

The electric excitation and the parameter excitation from mesh stiffness fluctuation are analyzed. The forced response equations of the drive system to the coupled excitations are presented. For the exciting frequencies far from and near natural frequencies, the forced responses of the drive system to the coupled excitations are investigated. Results show that the nonlinear forced responses of the drive system to the coupled excitations change periodically and unsteadily; the time period of the nonlinear forced responses depends on the frequencies of the electric excitation, the mesh parameter excitation, and the nonlinear natural frequencies of the drive system; in order to improve the dynamics performance of the drive system, the frequencies of the electric excitations should not be taken as integral multiple of the mesh parameter exciting frequency.

## 1. Introduction

Toroidal drive can transmit large torque in a very small size and is suitable for technical fields such as aviation and space flight [1–3]. As electrical and control techniques are utilized in mechanical engineering field widely, generalized composite drives become advancing edge of the mechanical science. So far, types of the generalized composite drives with integrated structure are still very limited.

The electromagnetic harmonic drive [4] and piezoelectric harmonic drive [5] are active drives in which the meshing forces between flexible gear and rigid one are controlled by electromagnetic force or piezoelectric one, and drive and power are integrated. Based on researching toroidal drive [6], the authors presented a kind of active generalized composite drive: electromechanical integrated toroidal drive. In the drive, the toroidal drive, power, and control are integrated [7].

**Figure 1:**
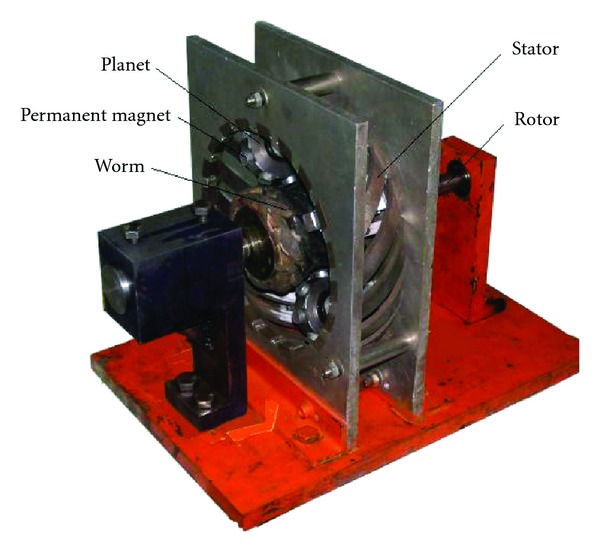
The electromechanical integrated toroidal drive.

The drive consists of four basic elements (Figure 1) (a) the central worm, (b) radially positioned planets, (c) a toroidal shaped stator, and (d) a rotor, which forms the central output shaft upon which the planets are mounted. The central worm is fixed and coils are mounted in helical grooves of its surface. The planets have permanent magnets instead of teeth. The *N* and *S* polar permanent magnets are mounted alternately on a planet. And the stator has helical permanent magnets instead of helical teeth. In the same manner as planet, the *N* and *S* polar helical permanent magnets are mounted alternately on the stator.

If a specific parameter relation is realized, *N* pole of one element will correspond to *S* pole of the other one all along. The attractive forces between *N* and *S* pole of the different elements are driving forces and the meshes without contact are realized. When the alternating voltage source is connected to the coils of the worm, a toroidal circular field is formed. It drives several planets to rotate about their own axial. And by means of magnetic forces between teeth of the planet and stator, the rotor is driven to rotate about its own axial. Thus, a power of low speed and large torque is output.

Compared with toroidal drive, the new drive is easy to produce, without wear, and does not need lubrication. It can be substituted for a servo system to simplify the structure of the existing electromechanical systems. Beside the above-mentioned fields that require compactness, the drive can be used in fields such as robots, which require accurate control. 

The electromechanical integrated toroidal drive consists of a mechanical system, an electrical system and a coupled part. The mechanical vibration may occur in the mechanical system, and the electrical current oscillation may occur in the electrical system. With the coupled part, the mechanical vibration and the electrical current oscillation will influence on each other. Hence, the drive system is an electromechanical coupled dynamics system. The electromechanical coupled dynamics was first proposed for the motor [8]. Then, the electromechanical coupled dynamics of the electromechanical system consisting of the motor and mechanical system driven by the motor was developed. An electromechanical coupled dynamics model of the electromechanical system consisting of the several motors and mechanical system driven by these motors was proposed. Using the model, the natural frequency of the electromechanical system is analyzed [9]. The authors investigated nonlinear forced response of electromechanical integrated toroidal drive to voltage excitations [10]. However, not only the electric excitation occurs, but also the parameter excitations from mesh stiffness fluctuations occur. The nonlinear forced responses of the drive system to the coupled excitations consisting of the electric excitation and the parameter excitations have not been investigated yet. These nonlinear forced responses have important influence on the operating performance of the drive system. To design, evaluate, and control dynamics behavior of the drive system effectively, the nonlinear forced responses of the drive system to the coupled excitations should be developed. 

In this paper, the electric excitation and the parameter excitation from mesh stiffness fluctuation are analyzed. The forced response equations of the drive system to the coupled excitations are presented. For the exciting frequencies far from and near natural frequencies, the forced responses of the drive system to the coupled excitations are investigated. The work can be used to predict the noise and dynamic load and are useful in maximizing the power density of the drive and reducing noise radiation.

## 2. Electric Excitation of the Drive System 

The magnetic energy storage of the electric system for the drive system is

(1) $$\begin{array}{c} W=\frac{1}{2}\sum_{i=1}^{n}\lambda_{is}I_{is}, \end{array}$$

where *λ*_*is*_ is magnetic linkage of the worm coils, *λ*_*is*_ = ∑_*j*=1_^*n*^ *L*_*ij*_ *I*_*is*_, *L*_*ij*_ is inductances of the *i*th phase worm coils, *I*_*is*_ is current of the *i*th phase worm coils, and *n* is phase number of the worm coils.

From (1), the electromagnetic torque on the planet is given as

(2) $$\begin{array}{c} T_{p}=-\frac{\partial W}{\partial \theta }=-\frac{1}{2}\sum_{j=1}^{n}\;\sum_{i=1}^{n}\frac{dL_{ij}}{d\theta }I_{is}I_{js}, \end{array}$$

where *θ* is the relative rotating angle between planet and worm. 

 The torque *T*_*p*_ consists of the static torque *T*_*p*0_ and the dynamic torque *δT*_*p*_. The current *I*_*is*_ consists of the static component *I*_*i*_ and the dynamic one *δi*_*i*_. The angle *θ* consists of the static angle *θ*_0_ and the dynamic angle *δθ*. The torque *T*_*p*_ is written in series form as

(3) Tp=Tp0+δTp=12∑j=1n ∑i=1n[(δLijδθ)θ=θ0IiIj+(δ2Lijδθ2)θ=θ0IiIjδθ    +(δ3Lijδθ3)θ=θ0IiIj(δθ)2+(δLijδθ)θ=θ0Iiδij    +(δLijδθ)θ=θ0Ijδii+(δLijδθ)θ=θ0Ii(δij)2    +(δLijδθ)θ=θ0Ij(δii)2+⋯].

If *δi*_*j*_ = *δi*_*i*_ = 0, from (3), one can give

(4) $$\begin{array}{c} T_{p0}=\frac{1}{2}\sum_{j=1}^{n}\;\sum_{i=1}^{n}{\left(\frac{\delta L_{ij}}{\delta \theta }\right)}_{\theta =\theta_{0}}I_{i}I_{j}, \end{array}$$

(5) δTp=12∑j=1n ∑i=1n[(δ2Lijδθ2)θ=θ0IiIjδθ        +(δ3Lijδθ3)θ=θ0IiIj(δθ)2+⋯].

Let Δ*F*_*wpi*_ denote the dynamic magnetic meshing force between a planet tooth and worm, Δ*F*_*wpi*_ = *δT*_*p*_/*R*. From (5), neglecting the high-order terms, one knows.

(6) $$\begin{array}{c} \Delta F_{wpi}=-\frac{R\delta \theta }{2R^{2}}\sum_{i=1}^{n}\;\sum_{j=1}^{n}\left[{\left(\frac{\delta^{2}L_{ij}}{\delta \theta^{2}}\right)}_{\theta =\theta_{0}}I_{i}I_{j}+{\left(\frac{\delta^{3}L_{ij}}{\delta \theta^{3}}\right)}_{\theta =\theta_{0}}I_{i}I_{j}\delta \theta \right]. \end{array}$$

Here, *ζ*_*wpi*_ = *R* *δθ*; it is the dynamic relative displacement between planet and worm. Thus, (6) can be changed into the following form:

(7) $$\begin{array}{c} \Delta F_{wpi}=\left(k_{wpi}+\Delta k_{wpi}\right)\zeta_{wpi}. \end{array}$$

Here, *k*_*wpi*_ = (1/2*R* ^2^)∑_*i*=1_^*n*^∑_*i*=1_^*n*^(*δ* ^2^ *L*_*ij*_/*δθ* ^2^)__*θ*=*θ*_0___ *I*_*is*_ *I*_*js*_, it is considered as linear electromagnetic mesh stiffness between a tooth of the planet and worm; Δ*k*_*wpi*_ = −(1/2*R* ^2^)∑_*i*=1_^*n*^∑_*j*=1_^*n*^(*δ* ^3^ *L*_*ij*_/*δθ* ^3^)_*θ*=*θ*_0__ *I*_*i*_ *I*_*j*_ *δθ*, it is nonlinear component of the electromagnetic mesh stiffness.

Equation (3) shows that current fluctuation can produce electromagnetic torque fluctuation. The torque fluctuation from current fluctuation can be considered as equivalent exciting torque Δ*T*_*e*_. Let the current periodically change as below:

(8) $$\begin{array}{c} \delta i_{i}=\delta i_{j}=\Delta I\cos\left(\omega_{e}t\right), \end{array}$$

where Δ*I* is the magnitude of the fluctuation current, *ω*_*e*_ is the frequency of the fluctuation current, and *t* is time.

Substituting (8) into (3), neglecting high-order terms, yields

(9) $$\begin{array}{c} \Delta T_{e}=\sum_{j=1}^{n}\;\sum_{i=1}^{n}{\left(\frac{\delta L_{ij}}{\delta \theta }\right)}_{\theta =\theta_{0}}I_{i}\Delta I\cos\omega_{e}t. \end{array}$$

Let Δ*F*_*e*_ denote the equivalent exciting force between a planet tooth and worm; thus

(10) $$\begin{array}{c} \Delta F_{e}=\frac{1}{R}\sum_{j=1}^{n}\;\sum_{i=1}^{n}{\left(\frac{\delta L_{ij}}{\delta \theta }\right)}_{\theta =\theta_{0}}I_{i}\Delta I\cos\omega_{e}t. \end{array}$$

## 3. Parametric Excitation from Stiffness Fluctuation

**Figure 2:**
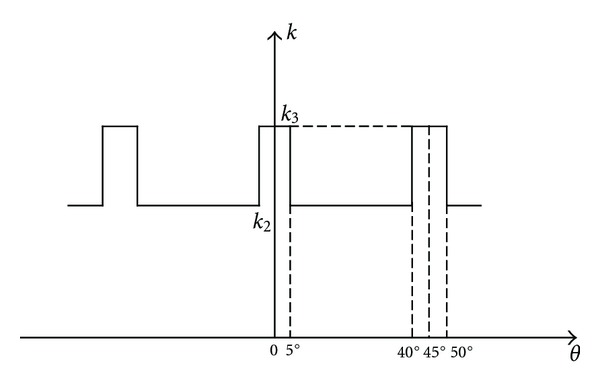
Changes of the mesh sifness.

In operation of the drive system, the number of meshing tooth pairs between the worm and the planets is variable. It can cause fluctuation of the mesh stiffness between them. It is dependent on the number *z*_1_ of planet teeth and the conditional face angles of the worm *ϕ*_*v*_. At *ϕ*_*v*_ = 100° and *z*_1_ = 8, the changes of mesh stiffness along with the mesh tooth pair number are shown in Figure 2. In Figure 2, a typical stiffness variation through a mesh cycle of the drive system is given. The mesh stiffness at more contact regions is higher than that at less contact regions. If the drive rotates at appreciable speed, this time-varying stiffness will be a major excitation source of the drive system.

The mesh stiffness between a planet and worm is considered to consist of its mean value $\overset{-}{k}$ and time-varying one Δ*k*(*t*). The average mesh stiffness between them through one periodic time can be given by

(11) $$\begin{array}{c} {\overset{-}{k}}_{wpi}=\frac{4}{\pi }\left\{\int_{0}^{\pi /36}k_{3}d\theta +\int_{\pi /36}^{2\pi /9}k_{2}d\theta +\int_{2\pi /9}^{\pi /4}k_{3}d\theta \right\}, \end{array}$$

where *k*_2_ = 2(*k*_*wpi*_ + Δ*k*_*wpi*_) and *k*_3_ = 3(*k*_*wpi*_ + Δ*k*_*wpi*_); they are two and three teeth mesh stiffness, respectively.

The periodical time-varying portion of the mesh stiffness can be defined in the Fourier series form as

(12) $$\begin{array}{c} \Delta k\left(t\right)=\sum_{n=1}^{\infty }\Delta k_{n}\cos n\omega_{p}t, \end{array}$$

where Δ*k*_*n*_ = (2/*l*)∫_0_^*l*^ *k*(*t*)*ω*_*p*_cos⁡⁡*nω*_*p*_ *tdt*, *l* is the period of the stiffness fluctuation. In Figure 2, *l* = *π*/4.

For worm and planet, the periodical time-varying portion of the mesh stiffness is

(13) Δknwpi=2l{∫0π/36(k3−k−wpi)ωpcos⁡⁡nωptdt   +∫π/362π/9(k2−k−wpi)ωpcos⁡⁡nωptdt   +∫2π/9π/4(k3−k−wpi)ωpcos⁡⁡nωptdt}=8π[(k3−k−wpi)(sin⁡⁡π36+sin⁡⁡π4−sin⁡⁡2π9)    +(k2−k−wpi)(sin⁡⁡2π9−sin⁡⁡π36)].

Substituting (13) into (12) yields

(14) $$\begin{array}{c} \Delta k_{wpi}\left(t\right)=\sum_{n=1}^{\infty }\Delta k_{nwpi}\cos n\omega t. \end{array}$$

## 4. Forced Response Equation to Coupled Excitation

**Figure 3:**
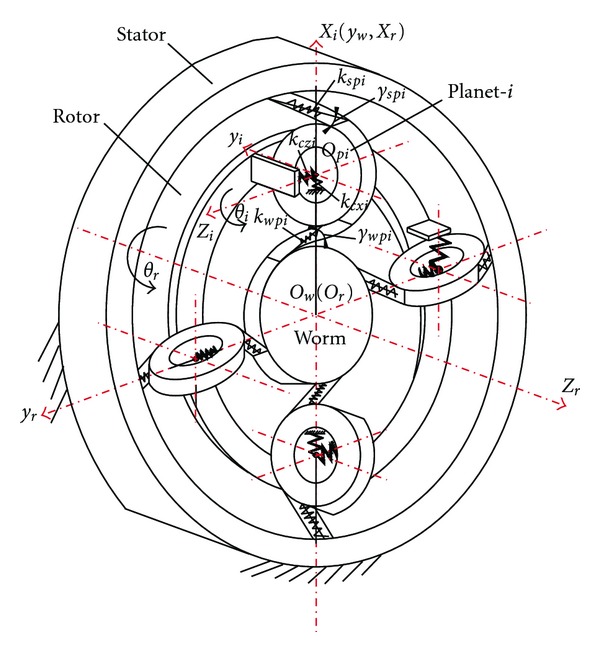
Dynamic model for a four-planet electromechanical integrated toroidal drive.

The dynamic model for the electromechanical integrated toroidal drive (see Figure 3) allows rotor and each planet to rotate about their own rotating axes and allows each planet to translate in *x*_*i*_ and *z*_*i*_ directions. The rotations are replaced by the corresponding translational mesh displacements as *u*_*j*_ = *r*_*j*_ *θ*_*j*_, *j* = 1,…, *m*, *r* (here, *m* is planet number, *θ*_*j*_ the rotation of planet or rotor, *r*_*j*_ is the rolling circle radius for planet and the radius of the circle passing through planet centers for the rotor). A displacement vector **q**_*j*_ and a mass matrix **m**_*j*_ are defined for each planet *j* as **q**_*j*_ = [*u*_*j*_ *x*_*j*_ *z*_*j*_]^*T*^ and **m**_*j*_ = Diag[*J*_*j*_/*r*_*j*_^2^ *m*_*j*_ *m*_*j*_]. Here, *J*_*j*_ and *m*_*j*_ are polar mass moment of inertia and mass for planet *j*, respectively. *M*_*r*_(*M*_*r*_ = *J*_*r*_/*r*_*r*_^2^) is equivalent mass of rotor corresponding to its displacement *u*_*r*_. Thus, the motion equations of the drive system are

(15) Jiri2u¨i+(kwpi+Δkwpi)pwpisin⁡⁡γwpi +ΔFesin⁡⁡γwpi−kspipspicos⁡⁡γspi=0,mix¨i+kcpxixi=0,miz¨i−(kwpi+Δkwpi)pwpicos⁡⁡γwpi −ΔFecos⁡⁡γwpi−kspipspisin⁡⁡γspi+kcpzipcpzi=0,Mru¨r−∑i=1mkcpzipcpzi=−Trrr (i=1  to  m),

where *p*_*wpi*_, *p*_*spi*_ and *p*_*cpzi*_ are relative displacements between planet-*i* and worm, stator, or rotor, respectively, *p*_*wpi*_ = *u*_*i*_sin⁡⁡*γ*_*wpi*_ − *z*_*i*_cos⁡⁡*γ*_*wpi*_, *p*_*spi*_ = −*u*_*i*_cos⁡⁡*γ*_*spi*_ − *z*_*i*_sin⁡⁡*γ*_*spi*_ and *p*_*cpzi*_ = *z*_*i*_ − *u*_*r*_. *γ*_*wpi*_ and *γ*_*spi*_ are lead angles at contact points between planet-*i* and worm or stator, respectively, tan⁡*γ*_*wpi*_ = 1/[*i*_*wp*_(*a*/*R* − 1)], and tan⁡*γ*_*spi*_ = 1/[*i*_*sp*_(*a*/*R* + 1)]. Here, *a* is center distance between worm and planet *R* is reference circle radius of planet *i*_*wp*_ and *i*_*sp*_ are speed ratios between planet and worm or stator, respectively. *k*_*wpi*_ and *k*_*spi*_ are mesh stiffness between planet-*i* and worm or stator, respectively, *k*_*czi*_ and *k*_*cxi*_ are planet support stiffness in *z*_*i*_ and *x*_*i*_ directions, respectively, and *T*_*r*_ is torque transmitted by rotor.

Equation (15) can be written in matrix form as

(16) $$\begin{array}{c} M\ddot{X}+KX=F+\varepsilon \Delta F+\varepsilon \Delta F_{e}, \end{array}$$

where **X** and **F** are displacement and static load vectors; respectively,

 
**X** = {**q**_1_ ⋯ ⋯ **q**_*m*_ *u*_*r*_}^*T*^,
 
**F** = {0 ⋯ ⋯ 0 −*T*_*r*_/*r*_*r*_}^*T*^;
 
**M** and **K** are mass and stiffness matrix, respectively,
 
**M** = diag⁡[**m**_1_ ⋯ ⋯ **m**_*m*_ *M*_*r*_],

(17) $$\begin{array}{c} K=\left[\begin{array}{cccccc} k_{wp1}+k_{sp1}+k_{cp1} & \begin{array}{cccc} & \cdot & \cdot & \cdot \end{array} & 0 & \begin{array}{ccc} \cdot & \cdot & \cdot \end{array} & 0 & k_{c1}\\ & \begin{array}{ccc} \cdot & & \\ & \cdot & \\ & & \cdot \end{array} & \begin{array}{c} \cdot \\ \cdot \\ \cdot \end{array} & \begin{array}{ccc} \cdot & \cdot & \cdot \\ \cdot & \cdot & \cdot \\ \cdot & \cdot & \cdot \end{array} & \begin{array}{c} \cdot \\ \cdot \\ \cdot \end{array} & \begin{array}{c} \cdot \\ \cdot \\ \cdot \end{array}\\ & & k_{wpi}+k_{spi}+k_{cpi} & \begin{array}{ccc} \cdot & \cdot & \cdot \end{array} & \cdot & k_{ci}\\ & & & \begin{array}{ccc} \cdot & & \\ & \cdot & \\ & & \cdot \end{array} & \begin{array}{c} \cdot \\ \cdot \\ \cdot \end{array} & \begin{array}{c} \cdot \\ \cdot \\ \cdot \end{array}\\ & & & & k_{wpm}+k_{spm}+k_{cpm} & k_{cm}\\ \text{symmetric} & & & & & \sum_{i=1}^{m}k_{czi} \end{array}\right],\\ k_{wpi}=k_{wpi}\left[\begin{array}{ccc} \text{si}{\text{n}}^{2}\gamma_{wpi} & 0 & -\cos\gamma_{wpi}\sin\gamma_{wpi}\\ 0 & 0 & 0\\ -\cos\gamma_{wpi}\sin\gamma_{wpi} & 0 & \mathrm{co}{\text{s}}^{2}\gamma_{wpi} \end{array}\right],\\ k_{spi}=k_{spi}\left[\begin{array}{ccc} \mathrm{co}{\text{s}}^{2}\gamma_{spi} & 0 & \sin\gamma_{spi}\cos\gamma_{spi}\\ 0 & 0 & 0\\ \sin\gamma_{spi}\cos\gamma_{spi} & 0 & \text{si}{\text{n}}^{2}\gamma_{spi} \end{array}\right],\\ k_{cpi}=\left[\begin{array}{ccc} 0 & 0 & 0\\ 0 & k_{cxi} & 0\\ 0 & 0 & k_{czi} \end{array}\right],\;\;k_{ci}={\left\{\begin{array}{ccc} 0 & 0 & -k_{czi} \end{array}\right\}}^{T}. \end{array}$$

Δ**F** is nonlinear component of the force vector, Δ**F**_*e*_ is equivalent force vector caused by current fluctuation: 

Consider

(18) $$\begin{array}{c} \Delta F=\left(Bu_{i}^{2}+Cu_{i}^{3}\right){\left[\begin{array}{cccccccc} -\sin\gamma_{wpi} & 0 & \cos\gamma_{wpi} & -\sin\gamma_{wpi} & 0 & \cos\gamma_{wpi} & \cdots & 0 \end{array}\right]}^{T},\;u_{i}=R\delta \theta ,\;\varepsilon =\frac{r}{R}, \end{array}$$

it is a disturbing parameter, *B* = *z*_1_∑_*i*=1_^*m*^∑_*j*=1_^*m*^ *A*tan⁡(*z*_1_ *θ* − *ϕ*_*v*_/*np*)*I*_*i*_ *I*_*j*_/2*Rr*, *C* = −*z*_1_^2^∑_*i*=1_^*m*^∑_*j*=1_^*m*^ *AI*_*i*_ *I*_*j*_/2*Rr*.

(19) $$\begin{array}{c} \Delta F_{e}=D\cos\left(\omega_{e}t\right){\left[\begin{array}{ccccccccccc} \sin\gamma_{wpi} & 0 & \cos\gamma_{wpi} & \sin\gamma_{wpi} & 0 & \cos\gamma_{wpi} & \sin\gamma_{wpi} & 0 & \cos\gamma_{wpi} & \cdots & 0 \end{array}\right]}^{T},\\ D=\frac{1}{2R}{\sum_{i=1}^{n}\;\sum_{j=1}^{n}\left(\frac{\partial L_{ij}}{\partial \theta }\right)}_{\theta =\theta_{0}}I_{i}\Delta I. \end{array}$$

The total displacement of the each component consists of the static displacement and the dynamic one. Hence, the displacement vector is

(20) $$\begin{array}{c} X=\overset{-}{X}+\Delta X, \end{array}$$

where $\overset{-}{X}$ and Δ**X** are static and dynamic displacement vectors; respectively,

(21) $$\begin{array}{c} \Delta X={\left\{\begin{array}{ccccc} \Delta q_{1} & \cdots & \cdots & \Delta q_{m} & \Delta u_{r} \end{array}\right\}}^{T},\\ \Delta q_{j}={\left[\begin{array}{ccc} \Delta u_{j} & \Delta x_{j} & \Delta z_{j} \end{array}\right]}^{T}. \end{array}$$

Considering the fluctuation of the mesh stiffness caused by the changes of the mesh tooth pair number, the stiffness matrix can be expressed as

(22) $$\begin{array}{c} K=\overset{-}{K}+\Delta K\left(t\right), \end{array}$$

where $\overset{-}{K}$ is the mean stiffness matrix, and Δ**K**(*t*) is time-varying stiffness matrix.

Substituting (20)–(22) into (16) yields

(23) $$\begin{array}{c} \overset{-}{K}\;\overset{-}{X}=F, \end{array}$$

(24) $$\begin{array}{c} M\Delta \ddot{X}+\overset{-}{K}\Delta X=\varepsilon \Delta F+\varepsilon \Delta F_{e}+\varepsilon \Delta F_{p}, \end{array}$$

where $\Delta F_{p}=-E\Delta K(t)\overset{-}{X}$; it is considered as the equivalent exciting force vector caused by mesh stiffness excitation; *E* = *R*/*r*.

Equation (24) is just the forced response equation of the drive system to coupled excitation.

## 5. Solution of the Nonlinear Forced Response Equation

### 5.1. Far from Natural Frequencies

First, we consider to resolve the nonlinear forced response equation of the drive system to electric excitation (let Δ**F**_*p*_ = 0).

For simplicity purposes, (24) should be transmitted into equations independent on each other. Then, (24) is changed into the following form:

(25) $$\begin{array}{c} \Delta {\ddot{X}}_{N}+{\overset{-}{K}}_{N}\Delta X_{N}=\varepsilon \Delta F_{N}+\varepsilon \Delta F_{eN}, \end{array}$$

where ${\overset{-}{K}}_{N}$ is the diagonal mean stiffness matrix, respectively. Δ**X**_*N*_ is transmitted dynamic displacement vector. Δ**F**_*eN*_ and Δ**F**_*N*_ are transmitted exciting force vectors of the forces Δ**F**_*e*_ and Δ**F**, respectively.

Matrix **K**_*N*_ and vector Δ**X**_*N*_ are given by **K**_*N*_ = **A**_*N*_^*T*^ **K** **A**_*N*_ and Δ**X**_*N*_ = **A**_*N*_^*T*^Δ**X**. The transmitted nonlinear force vector Δ**F**_*N*_ and transmitted equivalent exciting force vector Δ**F**_*eN*_ can be given as below:

(26) $$\begin{array}{c} \Delta F_{N}=A_{N}^{T}\Delta F=\left(Bu_{i}^{2}+Cu_{i}^{3}\right)\Delta P_{N},\\ \Delta F_{eN}=\varepsilon D\cos\left(\omega_{e}t\right)\Delta P_{eN}, \end{array}$$

where **A**_*N*_ is the mode matrix of (24):

(27) $$\begin{array}{c} A_{N}=\left[\begin{array}{cccc} A_{1}^{\left(1\right)} & A_{2}^{\left(1\right)} & \cdots & A_{m}^{\left(1\right)}\\ A_{1}^{\left(2\right)} & A_{2}^{\left(2\right)} & \cdots & A_{m}^{\left(2\right)}\\ \cdots & \cdots & \cdots & \cdots \\ A_{1}^{\left(m\right)} & A_{2}^{\left(m\right)} & \cdots & A_{m}^{\left(m\right)} \end{array}\right]. \end{array}$$

At *m* = 3, Δ**P**_*N*_ = [*P*_*N*1_ *P*_*N*2_ ⋯ *P*_*Ni*_ ⋯]^*T*^, **P**_*Ni*_ = −3*A*_*N*1_^*i*^sin⁡⁡*γ*_*wpi*_ + 3*A*_*N*3_^*i*^cos⁡⁡*γ*_*wpi*_  (*i* = 1,4,…, 3*m* − 2), *P*_*Ni*_ = 0 (*i* = 2,3,…, 3*m* + 1,  *i* ≠ 1,4,…, 3*m* − 2); Δ**P**_*eN*_ = [*P*_*eN*1_ 0 0 *P*_*eN*2_ 0 0 *P*_*eN*3_ 0 0 0]^*T*^, *P*_*Ne*_ = 3*A*_*N*1_^*i*^sin⁡⁡*γ*_*wpi*_ + 3*A*_*N*3_^*i*^cos⁡⁡*γ*_*wpi*_  (*i* = 1,4,…, 3*m* − 2), *P*_*Nei*_ = 0  (*i* = 2,3,…, 3*m* + 1, *i* ≠ 1,4,…, 3*m* − 2).

Let

(28) $$\begin{array}{c} \Delta X_{N}=X_{0}+\varepsilon X_{1}+\varepsilon^{2}X_{2}+\cdots , \end{array}$$

(29) $$\begin{array}{c} \omega_{i}^{2}=\omega_{0i}^{2}\left(1+\varepsilon \sigma_{1}^{i}+\varepsilon^{2}\sigma_{2}^{i}+\cdots \right)\;\left(i=1,2,3\right), \end{array}$$

where *ω*_*i*_ is natural frequency of *i*th order mode for the nonlinear drive system, and *ω*_0*i*_ is natural frequency of *i*th order rotational mode for the linear drive system.

Substituting (28) and (29) into (25), let sum of the coefficients with the same-order power of the parameter *ε* equal zero, following equations can be given

(30) x¨N0+ωi2xN0=0,x¨N1+ωi2xN1=−σ1x¨N0+PNBu0i2+PNCu0i3+Dcos⁡⁡(ωet)PNex¨N2+ωi2xN2=−σ1x¨N1−σ2x¨N0+PNB(2u0iu1i+σ1u0i2)        +PNC(3u0i2u1i+σ1u0i3)+Dσ1cos⁡⁡(ωet)PNe         ….

Here, initial conditions are *x*_*N*0_^*i*^(0) = *A*_*N*0_^*i*^, ${\dot{x}}_{N0}^{i}\left(0\right)=0$.

The solution of zero-order equation under the above initial conditions is

(31) $$\begin{array}{c} x_{0}^{i}=A_{0}^{i}\cos\omega_{i}t\;\left(i=1,2,3\right). \end{array}$$

Substituting (31) into the second equation of (30) yields

(32) x¨N11+ω12xN11=−σ11x¨N01+PN1Bu0i2+PN1Cu0i3+Dcos⁡⁡(ωet)PNe,x¨N12+ω22xN12=−σ12x¨N02+PN2Bu0i2+PN2Cu0i3+Dcos⁡⁡(ωet)PNe,x¨N13+ω32xN13=−σ13x¨N03+PN3Bu0i2+PN3Cu0i3+Dcos⁡⁡(ωet)PNe.

The rotational displacement *u*_*i*_ is

(33) $$\begin{array}{c} u_{i}=A_{N1}^{1}x_{Ni}^{1}+A_{N1}^{2}x_{Ni}^{2}+A_{N1}^{3}x_{Ni}^{3}. \end{array}$$

Substituting (33) into (30) yields

(34) x¨N11+ω12xN11 =−σ11x¨N01+PN1B(AN11xN01+AN12xN02+AN13xN03)2   +PN1C(AN11xN01+AN12xN02+AN13xN03)3   +Dcos⁡⁡(ωet)PNe1x¨N12+ω22xN12 =−σ12x¨N02+PN2B(AN11xN01+AN12xN02+AN13xN03)2   +PN2C(AN11xN01+AN12xN02+AN13xN03)3   +Dcos⁡⁡(ωet)PNe2,x¨N13+ω32xN13 =−σ13x¨N03+PN3B(AN11xN01+AN12xN02+AN13xN03)2   +PN3C(AN11xN01+AN12xN02+AN13xN03)3   +Dcos⁡⁡(ωet)PNe3.

In order to remove secular item, let

(35) σ11=−PN1Cω12  ×[34(AN11AN01)3+32(AN12AN02)2AN11AN01     +32(AN13AN03)2AN11AN01],σ12=−PN2Cω22  ×[34(AN12AN02)3+32(AN11AN01)2AN12AN02     +32(AN13AN03)2AN12AN02],σ13=−PN3Cω32  ×[34(AN13AN03)3+32(AN12AN02)2AN13AN03     +32(AN11AN01)2AN13AN03].

Substituting (35) into (34), the solutions of the first-order equations can be obtained. As the equations of the solutions are relatively complicated, it is not given here.

In a same manner, the solution of *n*th order equation can be obtained as well. Substituting these solutions into (28) and (29), the solutions of the regular nonlinear forced response equations and natural frequencies of the drive system can be given. Then, the real solutions of the nonlinear forced responses can be calculated as below:

(36) $$\begin{array}{c} \Delta X=A_{N}\Delta X_{N}. \end{array}$$

### 5.2. Near Natural Frequencies

When the exciting frequency is near natural frequency, the nonlinear forced response equation can be resolved as below. Considering damping of the drive system, (25) can be changed into

(37) $$\begin{array}{c} \Delta {\ddot{X}}_{N}+C_{N}\Delta {\dot{X}}_{N}+K_{N}\Delta X_{N}=\varepsilon \Delta F_{N}+\varepsilon \Delta F_{eN}, \end{array}$$

where **C**_*N*_ is regular damping matrix, **C**_*N*_ = **A**_*N*_^*T*^ **C** **A**_*N*_ = diag⁡[*C*_*N*1_ *C*_*N*2_ *C*_*N*3_ *C*_*N*4_] (**C** is damping matrix). *C*_*Ni*_ = 2*ζω*_*i*_, *ζ* is relative damping coefficient.

When exciting frequency is near natural frequency, the exciting frequency can be written as

(38) $$\begin{array}{c} \omega_{ei}^{2}=\omega_{0i}^{2}\left(1+\varepsilon \sigma_{1}^{i}\right)\;\left(i=1,2,3\right). \end{array}$$

Substituting (38) and (28) into (37), let sum of the coefficients with the same-order power of the parameter *ε* equal zero, the following equations can be given:

(39) X¨0+KNX0=0,X¨1+KNX1=−σ1iX¨0−2ζ1X˙0        +ΔPN(Biui2+Ciui3)+ΔPeNDicos⁡⁡(ωeit+θ)         ⋯,

where *C*_*Ni*_ = 2*ζω*_*i*_,

(40) $$\begin{array}{c} \zeta =\varepsilon \zeta_{1},\\ \omega_{ei}^{2}=\omega_{i}^{2}\left(1+\varepsilon \sigma_{1}\right),\\ \Delta x_{Ni}=x_{N0i}+\varepsilon x_{N1i},\\ B=B_{i}\omega_{i}^{2},\\ C=C_{i}\omega_{i}^{2},\\ D=D_{i}\omega_{i}^{2}. \end{array}$$

The solution of zero-order equation under the above initial conditions is

(41) $$\begin{array}{c} x_{N0}^{i}=A_{N0}^{i}\cos\omega_{ei}t\;\left(i=1,2,3\right). \end{array}$$

Substituting (41) into the second equation of (43) yields

(42) x¨N11+ωe12xN11=−σ1x¨N01−2ζ1x˙N01+PN1B1u012+PN1C1u013+D1PNe1cos⁡⁡(ωe1t+θ),x¨N12+ωe22xN12=−σ1x¨N02−2ζ1x˙N02+PN2B2u012+PN2C2u013+D2PNe2cos⁡⁡(ωe2t+θ),x¨N13+ωe32xN13=−σ1x¨N03−2ζ1x˙N03+PN3B3u012+PN3C3u013+D3PNe3cos⁡⁡(ωe3t+θ).

Substituting rotational displacement *u*_*i*_ = *A*_*N*1_^1^ *x*_*Ni*_^1^ + *A*_*N*1_^2^ *x*_*Ni*_^2^ + *A*_*N*1_^3^ *x*_*Ni*_^3^ into (42) yields

(43) x¨N11+ωe12xN11 =−σ1x¨N01−2ζ1x˙N01+PN1B1(AN11xN01+AN12xN02+AN13xN03)2   +PN1C1(AN11xN01+AN12xN02+AN13xN03)3   +D1PNe1(cos⁡⁡ωe1tcos⁡⁡θ+sin⁡⁡ωe1tsin⁡⁡θ),x¨N12+ωe22xN12 =−σ1x¨N02−2ζ1x˙N02+PN2B2(AN11xN01+AN12xN02+AN13xN03)2   +PN2C2(AN11xN01+AN12xN02+AN13xN03)3   +D2PNe2(cos⁡⁡ωe2tcos⁡⁡θ+sin⁡⁡ωe2tsin⁡⁡θ),x¨N13+ωe32xN13 =−σ1x¨N03−2ζ1x˙N03+PN3B3(AN11xN01+AN12xN02+AN13xN03)2   +PN3C3(AN11xN01+AN12xN02+AN13xN03)3   +D3PNe3(cos⁡⁡ωe3tcos⁡⁡θ+sin⁡⁡ωe3tsin⁡⁡θ).

In order to remove secular item, let

(44) $$\begin{array}{c} \sigma_{1}^{1}A_{N0}^{1}+P_{N1}C_{1}A_{N0}^{1}P_{1}^{\prime }+D_{1}P_{Ne1}\cos\theta =0,\\ 2\zeta_{1}A_{N0}^{1}+D_{1}P_{Ne1}\sin\theta =0, \end{array}$$

where *P*_1_′ = (1/*A*_*N*0_^1^)[(3/4)(*A*_*N*1_^1^ *A*_*N*0_^1^)^3^ + (3/2)(*A*_*N*1_^2^ *A*_*N*0_^2^)^2^ *A*_*N*1_^1^ *A*_*N*0_^1^ + (3/2)(*A*_*N*1_^3^ *A*_*N*0_^3^)^2^ *A*_*N*1_^1^ *A*_*N*0_^1^].

From (44), it is known that

(45) $$\begin{array}{c} {\left(\sigma_{1}^{1}+P_{N1}C_{1}P_{1}^{\prime }\right)}^{2}+{\left(2\zeta_{1}\right)}^{2}={\left(\frac{D_{1}P_{Ne1}}{A_{N0}^{1}}\right)}^{2}. \end{array}$$

Thus

(46) si2=1−εPNiCiPi′−2ζ2±(Di′AN0i)2−4ζ2(1−εPNiCiPi′−ζ2),

where *s*_*i*_ = *ω*_*ei*_/*ω*_*i*_ and *D*_*i*_′ = *εD*_*i*_ *P*_*Nei*_.

From (46), the changes of the nonlinear vibrating magnitudes along with exciting frequencies can be given.

## 6. Results and Discussions

**Figure 4:**
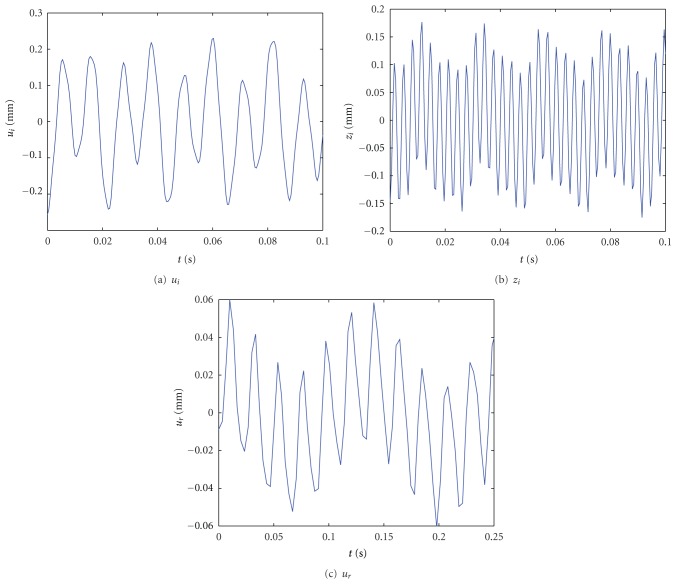
Nonlinear forced vibration to coupled excitations far from natural frequencies.

When exciting frequency is far from natural frequency, from the above equations, the nonlinear forced vibrations for the drive system are analyzed. The parameters of the numerical example are shown in Table 1.  Figure 4 shows changes of the forced vibrations of the transmitted variables along with nonlinear parameter *ε*. From Figure 4, the following are known.

1. Under the coupled excitations, the nonlinear forced responses of the drive system change periodically and unsteadily. The time period of the nonlinear forced responses depends on the frequencies of the electric excitation the mesh parameter excitation, and the nonlinear natural frequencies of the drive system.
2. The vibrating amplitudes of the nonlinear forced responses of the drive system to the coupled excitations are larger than those of the nonlinear forced responses to the single excitation. The vibrating amplitudes of the nonlinear forced responses of the planet are larger than that of the rotor. It is because the exciting frequencies are near to the vibrating frequency of the planet modes.
3. The vibrating amplitudes of the tangent vibration for the planet are larger than that of the axial vibration for the planet, and the frequency of the tangent vibration for the planet is smaller than that of the axial vibration for the planet.
4. The unstable periodic vibrations are harmful for the drive operation. In order to increase the dynamics performance of the drive system, the electric excitations should be avoided or the frequencies of the electric excitations should not be taken as integral multiple of the mesh parameter exciting frequency.

**Figure 5:**
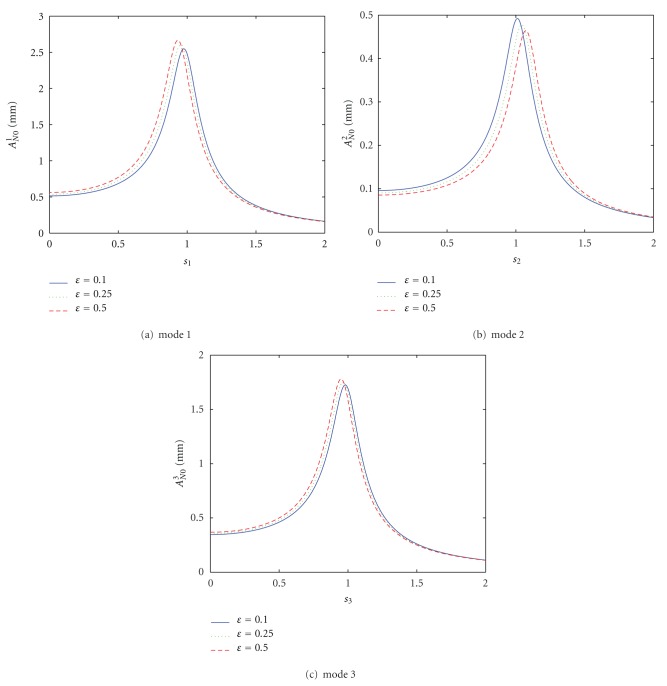
Nonlinear forced vibration to coupled excitations near natural frequencies.

When exciting frequency is near natural frequency, changes of the nonlinear vibrating magnitudes along with exciting frequencies and the drive parameters are given in Figure 5. From Figure 5, the following are known.

1. As exciting frequency increase, the vibrating magnitudes of all the modes increases, and at point *s*_*i*_ = *ω*_*ei*_/*ω*_*i*_ ≈ 1, the vibrating magnitudes get to the maximum, and then they decrease with increasing exciting frequency. For different modes, the previously mentioned curves bend toward the direction of the exciting frequency increase. The results are typical nonlinear character of the drive system.
2. For the first and third modes, as nonlinear parameter *ε* increases, their vibrating magnitudes increase. For the second mode, as nonlinear parameter *ε* increases, its vibrating magnitude decreases. The vibrating magnitudes are large for the first and third modes and small for the second mode. The vibrating magnitude for the first mode is larger than that for the third mode.
3. For a given exciting frequency, the vibrating amplitudes of the nonlinear forced responses of the drive system to the coupled excitations for modes 1 and 3 are larger than those of the nonlinear forced responses to the single excitation; for mode 2, the vibrating amplitude of the nonlinear forced responses of the drive system to the coupled excitations is smaller than that of the nonlinear forced responses to the single excitation.
4. Under the coupled excitations, the nonlinearity has obvious effects on the relationship between the frequency and the amplitudes; it should be considered.

## 7. Conclusions

In this paper, the electric excitation and the parameter excitation from mesh stiffness fluctuation are analyzed. The forced response equations of the drive system to the coupled excitations are presented. For the exciting frequencies far from and near to natural frequencies, the forced responses of the drive system to the coupled excitations are investigated. Results show the following.

1. Under the coupled excitations, the nonlinear forced responses of the drive system change periodically and unsteadily.
2. The time period of the nonlinear forced responses depends on the frequencies of the electric excitation the mesh parameter excitation, and the nonlinear natural frequencies of the drive system.
3. In order to improve the dynamics performance of the drive system, the frequencies of the electric excitations should not be taken as integral multiple of the mesh parameter exciting frequency.

## Figures and Tables

**Table 1:** Parameters of the example system.

*ε*	*ω*_*e*_ (rad/s)	*a*/*R*	*i*_wpi_	*NI*_*s*_ (A)	*r* (mm)	*I* (A)	*C*	*D*	*R* (mm)	*z*_1_	*L* (H)
0.25	1000	2	8	100	25	180	8707.2	−0.00066	100	8	1 × 10^−3^
